# Mitochondria-Endoplasmic Reticulum Contacts: The Promising Regulators in Diabetic Cardiomyopathy

**DOI:** 10.1155/2022/2531458

**Published:** 2022-04-11

**Authors:** Yan Chen, Yanguo Xin, Yue Cheng, Xiaojing Liu

**Affiliations:** ^1^Laboratory of Cardiovascular Diseases, Regenerative Medicine Research Center, West China Hospital, Sichuan University, Chengdu 610041, China; ^2^Department of Cardiology, West China Hospital, Sichuan University, Chengdu 610041, China

## Abstract

Diabetic cardiomyopathy (DCM), as a serious complication of diabetes, causes structural and functional abnormalities of the heart and eventually progresses to heart failure. Currently, there is no specific treatment for DCM. Studies have proved that mitochondrial dysfunction and endoplasmic reticulum (ER) stress are key factors for the development and progression of DCM. The mitochondria-associated ER membranes (MAMs) are a unique domain formed by physical contacts between mitochondria and ER and mediate organelle communication. Under high glucose conditions, changes in the distance and composition of MAMs lead to abnormal intracellular signal transduction, which will affect the physiological function of MAMs, such as alter the Ca^2+^ homeostasis in cardiomyocytes, and lead to mitochondrial dysfunction and abnormal apoptosis. Therefore, the dysfunction of MAMs is closely related to the pathogenesis of DCM. In this review, we summarized the evidence for the role of MAMs in DCM and described that MAMs participated directly or indirectly in the regulation of the pathophysiological process of DCM via the regulation of Ca^2+^ signaling, mitochondrial dynamics, ER stress, autophagy, and inflammation. Finally, we discussed the clinical transformation prospects and technical limitations of MAMs-associated proteins (such as MFN2, FUNDC1, and GSK3*β*) as potential therapeutic targets for DCM.

## 1. Introduction

Diabetes mellitus (DM) is a metabolic disorder characterized by insulin resistance, hyperglycemia, and hyperlipidemia. DM-related complications affect multiple organs. DM causes cardiomyopathy and increases the risk of heart failure, independent of the traditional risk factors, such as hypertension, coronary artery disease, and valvular heart disease, which is clinically known as diabetic cardiomyopathy (DCM) [1]. DCM is a common complication of DM with a poor outcome [2]. The development of DCM is a gradual process. Early-stage DCM is characterized by structural and functional abnormalities, including cardiac hypertrophy, increased myocardial stiffness, myocardial fibrosis, and diastolic dysfunction. As DCM progresses, these abnormalities may lead to heart failure [3]. Effective therapeutic measures for DCM, especially specific therapeutic drugs, are lacking.

The pathophysiology of DCM is multifactorial. Several pathophysiological mechanisms, including hyperglycemia, energy metabolism disturbances, and inflammation, have been proposed. In addition, mitochondrial dysfunction and ER stress are known to be crucial factors in the development and progression of DCM [3] [4]. Both mitochondria and the ER are vital organs in cells, with the functions of mitochondria and the ER highly interconnected. Mitochondria are highly dynamic structures, with their morphologies and compositions constantly changing to meet cellular requirements [5]. They are connected to membranous organelles, including the ER. The contact sites between mitochondria and the ER are known as mitochondria-associated ER membranes (MAMs). MAMs are unique domains that mediate the tight connection between mitochondria and the ER [6]. MAMs serve as the essential hub for intraorganelle communication in cells.

Dysregulation of MAMs contributes to the etiology of many diseases. An increasing amount of data suggests that MAMs play significant roles in pathological conditions, including neurodegenerative disorders [7], metabolic diseases [8], and cardiovascular diseases [9], by regulating intraorganelle lipid exchange, calcium (Ca^2+^) transfer, mitochondrial dynamics, ER stress, inflammation, autophagy, and apoptosis [10] and that these processes are involved in DCM. As such, MAMs are closely related to the pathophysiological mechanism of DCM.

In DM, destruction of the mitochondria-ER contacts in cardiomyocytes leads to dysfunction of several molecular pathways, thereby inducing cell death and cardiac dysfunction [11]. For example, knockout of MAM components such as Mitofusin 2 (MFN2) [12], inositol 1,4,5-triphosphate receptors (IP3R1), or cyclophilin D (CypD) [13] will interrupt MAM integrity and induce insulin resistance. The latter is closely related to mitochondrial dysfunction, ER stress, and altered Ca^2+^ homeostasis in DCM [11, 14]. Excessive mitochondria-ER coupling also has adverse effects, with recent research showing that enhancement of the MAM formation is involved in metabolic pathologies, such as insulin resistance and diabetes [15]. Therefore, a moderate but not excessive level of MAM formation is necessary for cell function.

We hypothesized that MAMs may be the promising regulator in the physiopathology of DCM. In the present review, we first introduce the structure and components of MAMs. We then we discuss the role of MAMs in DCM and summarize their emerging clinical use as potential biomarkers and therapeutic targets for DCM.

## 2. Structure of Mitochondria-ER Contacts

In 1990, Vance et al. described the components of membrane coupling between the ER and mitochondria in rat liver [16]. Since then, researchers have used a combination of electron microscopy and cell fluorescence microscopy to reveal the microstructure of MAMs. Using electronic tomography, researchers showed that the ER and mitochondria are connected by tethers and that the distance between the ER and mitochondria is approximately 25 nm for rough ER and approximately 10 nm for smooth ER [17]. Observations using a wide-field digital 3D deconvolution microscope showed that about 20% of the ER is in direct contact with the mitochondrial surface in MAMs [18].

It is important to consider the frequency and spacing between mitochondria and the ER in MAMs because they are dynamic structures, and the contacts and distance between mitochondria and the ER can vary widely under different cellular physiological conditions. The dynamic and flexible characteristics of MAMs results in a highly variable MAM composition. A proteomic study of MAM components showed that as many as 1212 proteins are localized in MAMs [19]. After excluding contaminated samples, approximately 75 proteins, including sorting proteins, chaperone proteins, and protein kinases, were shown to be associated with MAMs [20, 21].

**Figure 1:**
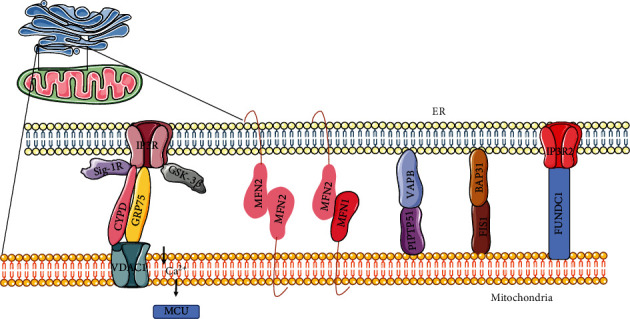
A core subset of mitochondria-ER tethering complexes in mammalian cells. IP3R, an ER protein, interacts OMM-localized protein VDAC1 via GRP75. The Sig-1R and GSK3*β* interact with IP3Rs-GRP75-VDAC1 complex. MFN2 on the ER tethers the ER to mitochondria by forming complexes with MFN1 or MFN2 on the mitochondrial surface. An ER resident protein, VAPB interacts with the OMM protein PTPIP51.BAP31, an ER protein, interacts with the mitochondria FIS1. The ER resident IP3R2 interacts the mitochondrial protein FUNDC1. IP3R, inositol 1,4,5-triphosphate receptor; VDAC1, voltage-dependent anion-selective channel; GRP75, glucose-regulated protein 75; MFN2, Mitofusin 2; MFN1, Mitofusin 2; VAPB, vesicle-associated membrane protein-associated protein B; PTPIP51, protein tyrosine phosphatase interacting protein 51; BAP31, B-cell receptor-associated protein 31; FIS1, mitochondrial fission 1; FUNDC1, FUN14 domain containing 1.

MAMs have a unique structure, with a large number of proteins with various functions (Table 1).  Figure 1 shows the key tethering protein complexes between mitochondria and the ER in mammalian cells (Figure 1). (I) The inositol 1,4,5-triphosphate receptor-glucose-regulated protein 75-voltage-dependent anion-selective channel 1 (IP3R-GRP75-VDAC1) complex is composed of IP3Rs in the ER and VDAC1 at the outer mitochondrial membrane (OMM). GRP75 is a chaperone. It bridges the IP3R and VDAC1 to maintain conformational stability and forms an ER-mitochondrial Ca^2+^ tunnel [22]. (II) MFN2 ethers the ER to mitochondria by forming heterotypic or homotypic complexes with MFN1 or MFN2 on the mitochondrial surface. These complexes mediate mitochondrial dynamics and mitochondrial Ca^2+^ uptake [23]. (III) The vesicle-associated membrane protein-associated protein B-protein tyrosine phosphatase interacting protein 51 (VAPB-PTPIP51) complex consists of the OMM protein PTPIP51 and the ER resident protein VAPB, which maintains ER-mitochondrial Ca^2+^ homeostasis [24]. (IV) The B-cell receptor-associated protein 31-mitochondrial fission 1 (BAP31-FIS1) complex is formed via the interaction of ER-localized BAP31 and mitochondrial FIS1, which participates in mitochondrial dynamics and cell apoptosis [25]. (V) Recently, another important protein bridge was reported, with the bridge formed through the interaction of the OMM human protein FUN14 domain-containing 1 (FUNDC1) with the IP3R2, which modulates the Ca^2+^ transport in the ER into mitochondria and maintains mitochondrial dynamics [26]. Various regulators modulate these tether complexes. For example, the IP3Rs-GRP75-VDAC1 complex is regulated by CypD, Sigma-1 receptor (Sig-1R), glycogen synthase kinase-3*β* (GSK-3*β*) and pyruvate dehydrogenase kinase 4 [27, 28].

Abnormality in MAMs resident proteins and regulators may be associated with the pathology of various diseases. For example, the insulin signaling protein AKT is located in MAMs, interacting with promyelocytic leukemia (PML)-protein phosphatase 2A (PP2A)-IP3R to form a large molecular complex in MAMs and controlled ER Ca^2+^ release [29]. Recently, a few of studies support that the presence of ATK regulate MAMs integrity [29–31]. Under DM condition or insulin resistance, an increased phosphorylation of AKT was found in the mice, which induces disruption of MAMs and may be the critical cause of Ca^2+^ disorder and mitochondrial dysfunction in DCM. Additionally, the key MAM proteins, VDAC1, CypD, and PACS2, decreased significantly in insulin-resistant mice [30], which suggest that MAMs are overtly altered under DCM condition; targeted regulation of these MAMs is expected to alleviate the disease to a certain extent.

In the future, more tethers and regulatory proteins will be identified between the mitochondria and the ER, and studies are required to investigate the alteration of MAMs resident proteins contributing to the pathogenesis of DCM.

## 3. The Role of MAMs in DCM

In this section, we consider MAM proteins and their regulators that play central roles in ER-mitochondrial crosstalk (Table 1). In DM condition, destruction of MAMs integrity in cardiomyocytes directly or indirectly causes imbalances in several biological processes, which may explain the metabolic abnormalities in DCM.

### 3.1. MAMs Regulate Ca^2+^ Transfer in DCM

Mitochondria and the ER (sarcoplasmic reticulum in muscle cells) are the main organelles that regulate Ca^2+^ homeostasis in cells. Mitochondrial Ca^2+^ uptake is dependent on close binding of MAM-mediated ER to mitochondria [32]. The sarcoplasmic reticulum (SR)/ER Ca^2+^ ATPase (SERCA) is greatly enriched in MAMs, which is an ER membrane influx transporter through which Ca^2+^ from the cytoplasm is transported to the ER [33]. Ca^2+^ from the ER is transferred to mitochondria through MAMs [22] and then enters the mitochondrial matrix through the mitochondrial calcium uniporters (MCUs) [34]. In this process, the distance of the ER and mitochondria is a key parameter for Ca^2+^ transport. At shorter distances, Ca^2+^ transport is more efficient. Conversely, Ca^2+^ transport becomes less efficient as the distance increases [35]. Among MAM tethering protein complexes, IP3R-GRP75-VDAC1 is the most important tethering complex mediating Ca^2+^ efficient transfer in MAMs. The releases of Ca^2+^ from the ER to the OMM through the IP3R-GRP75-VDAC1 channel leads to local inflow of Ca^2+^ in the mitochondrial intermembrane space and the formation of a microdomain of high Ca^2+^ close to MCUs, which facilitates the mitochondrial Ca^2+^ uptake by MCU [36]. As shown in recent studies, the IP3R-GRP75-VDAC1 complex also acts as a molecular scaffold for several other proteins, such as glycogen synthase kinase-3*β* (GSK-3*β*) [37], protein kinase B (PKB)/Akt [38], and promyelocytic leukemia (PML) [29]. These proteins are crucial for the fine tuning of Ca^2+^ signaling via IP3R-GRP75-VDAC1 axis (Table 1).

**Figure 2:**
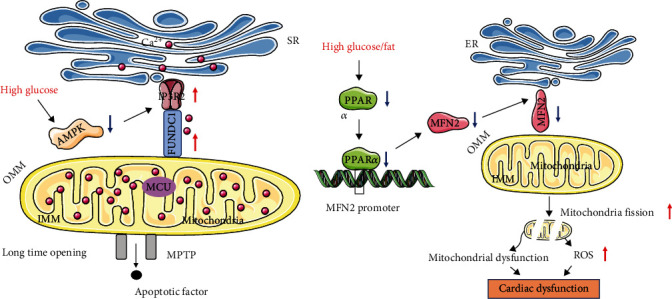
(a). The role of FUNDC1 and MFN2 in DCM. Under high glucose condition, FUNDC1 is upregulated by AMPK inactivation. The upregulated FUNDC1 increases MAMs formation by interacts with IP3R2. The increased MAMs contribute to the increased mitochondrial Ca^2+^ uptake, which induce the long-time opening of mPTP and trigger cell apoptosis. (b). High glucose or high fat reduces the PPAR*α* expression. PPAR*α* directly binding to MFN2 promoter and decreases the expression of MFN2. The downregulation of MFN2 induces mitochondrial fission and leads to the ROS production, which cause mitochondrial dysfunction and cardiac dysfunction. MFN2, Mitofusin 2; FUNDC1, FUN14 domain containing 1; AMPK, AMP-activated protein kinase; IP3R2, inositol 1,4,5-triphosphate receptor 2; PPAR*α*, peroxisome proliferator-activated receptor alpha.

Due to its role in excitation-contraction coupling in muscle tissue, Ca^2+^ signaling is crucial for heart function, and Ca^2+^ disturbance mediated by destruction of the integrity of MAMs is closely associated with cardiac dysfunction in DCM. Interaction between several cellular Ca^2+^ transporter complexes controls the diastolic and systolic functions of the myocardium. During diastole, myocardial relaxation occurs due to reuptake of Ca^2+^ into the SR via SERCA2a [14]. In diabetes, reduced activity and expression of SERCA2a in cardiomyocytes result in altered Ca^2+^ handling, which leads to impairment of left ventricular diastolic function and the development of DCM [39]. Disordered Ca^2+^ transfer through MAMs is the crucial cause for DCM. In the early stage of DCM, a reduced formation of the IP3R-GRP75-VDAC1 Ca^2+^ channeling complex and decreased IP3R-stimulated Ca^2+^ transfer to mitochondria trigger mitochondrial dysfunction [40]. A decrease in the ER-mitochondrial Ca^2+^ transfer leads to insufficient mitochondrial bioenergetics to match the energy demand for normal heart contraction [40]. However, inconsistent finding on the role of Ca^2+^ transport in DCM has been reported. Hu et al. reported that a high glucose level enhances the connections between the SR and mitochondria and increases the efficiency of Ca^2+^ transfer, which causes Ca^2+^ overload in mitochondria [41]. Wu's study showed that FUNDC1 is important in mediating MAMs formation in diabetes, with high glucose increasing FUNDC1 levels, IP3R2 levels, and MAMs formation, resulting in increased Ca^2+^ levels, mitochondrial dysfunction, and deterioration of cardiac function [11]. High glucose increases MAMs-associated FUNDC1 levels by downregulating AMP-activated protein kinase (AMPK). And binding of FUNDC1 to the IP3R2 inhibits IP3R2 ubiquitination and proteasome-mediated degradation, which promotes contacts between the ER and mitochondria, resulting in increased Ca^2+^ transport and a decline in the mitochondrial membrane potential [11]. These events trigger long-term mitochondrial permeability transition pore (mPTP) opening and cell apoptosis by promoting mitochondrial Ca^2+^ uptake [42] (Figure 2(a)). We hypothesize that this inconsistent result of Ca^2+^ homeostasis in DCM is due to different severities and stages of the disease.

According to the literature, MFN2 regulates Ca^2+^ transfer in high glucose conditions, with high glucose upregulating the expression of MFN2, decreasing the distance between the ER and mitochondria, and increasing mitochondrial Ca^2+^ uptake in atrial cardiomyocytes [43]. And knocking down MFN2 significantly disrupted ER-mitochondrial tethering and decreased the Ca^2+^ transportation, thereby preventing mitochondrial dysfunction and cell death [43]. However, there is still controversy regarding the role of MFN2. Filadi et al. demonstrated that MFN2 ablation or silencing increased the ER-mitochondrial contacts, enhancing Ca^2+^ transfer between the two organelles, with the function of MFN2 similar to that of a tethered antagonist [44]. The use of different physiological conditions, cell types, and experimental approaches may explain the discord in the literature on the role of MFN2 in DCM. However, the detailed mechanism of how MFN2 participates in the Ca^2+^ transport in DCM is still unclear. In addition to mediating the spatial distance from the ER to mitochondria and affecting the transport efficiency of Ca^2+^, it is interesting to explore whether it forms a complex with Ca^2+^ channel proteins. The exact mechanism of MFN2 in DCM deserves to be further investigated.

### 3.2. MAMs Modulate Mitochondrial Dynamics in DCM

Mitochondria are highly dynamic organelles, and balancing mitochondrial dynamics is essential to maintain heart function in response to metabolic or environmental stresses [5]. Various GTPases are involved in the regulation of mitochondrial dynamics, including dynamin-like GTPases optic atrophy 1 (OPA1), which plays a role in inner mitochondrial membrane (IMM) fusion, together with MFN1 [45]. FUNDC1 interacts with OPA1 to coordinate mitochondrial fusion [46]. Under normal physiological conditions, FUNDC1 anchor OPA1 to the inner surface of OMM through its charged lysine residue. Mitochondrial stresses disrupt the connection of FUNDC1 and OPA1 and induce OPA1 cleavage or even degradation, which promotes mitochondrial fission [46]. Extracellular hyperglycemia and metabolic dysregulation create an energy stress in DCM, which reduce the interaction of OPA1 and FUNDC1, increasing mitochondrial fragmentation. MFN1 and MFN2 are crucial for the fusion of OMM [23], with deletion of MFN1 or MFN2 reduces the mitochondrial fusion rate [47]. In DCM, downregulation of MFN2 contributes to unbalanced mitochondrial dynamics and mitochondrial dysfunction [48]. The downregulation of MFN2 is partly attributed to the decreased expression of the peroxisome proliferator-activated receptor alpha (PPAR*α*) caused by the lipid metabolism disorder in DCM [49] (Figure 2(b)). Reconstitution of MFN2 improves mitochondrial function by promoting mitochondrial fusion [48]. Accordingly, modulation of mitochondrial dynamics by regulating MFN2 might be a potentially effective target for DCM treatment.

Dynamin-related protein 1 (DRP1) plays a central role in mitochondrial fission [18]. DRP1 is a cytoplasmic protein that can be recruited from the cytosol to the OMM, which is a critical step in the fission process. Friedman et al. showed that MAM is an important platform for mitochondrial fission. In their study, they demonstrated oligomerization and translocation of DRP1 to MAMs, where it induced fission events [18]. Subsequent studies revealed the mechanism that MAMs participate in initial mitochondria contraction. This mechanism involves ER-localized inverted formin 2 (INF2) inducing actin polymerization, which promotes MAMs formation, facilitating Ca^2+^ transfer from ER to mitochondria, followed by IMM contraction and initial mitochondrial constriction. This is followed by DRP1-driven secondary constriction, which completes the fission process [50, 51].

In patients diagnosed with diabetes, myocardial contractile dysfunction is closely associated with mitochondria fission. In diabetes, increased expression of DRP1 initiates mitochondrial fission. Conversely, decreased expression of DRP1 decrease alleviates mitochondrial dysfunction and cardiac dysfunction [52]. Mechanically, lipid overload decreased NAD^+^ levels and increased the acetylation of DRP1 at a specific lysine residue (K642). A DRP1 point mutation, K642E, appears to reverse the impact of lipid toxicity. Excessively activation of DRP1 results in DRP1 translocated to mitochondria, induces mPTP and apoptosis, and compromises cardiomyocyte contractile function via VDAC1 [53].

Several proteins have been shown to regulate DRP1 activity at MAMs. Under normal physiological condition, mitochondrial FIS1, mitochondrial fission factor, and mitochondrial dynamics proteins of 49/51 kDa have been reported to recruit DRP1 during mitochondrial fission [54, 55]. Regulating DRP1 activity by handing these molecules at contact site may be an appropriate strategy to prevent the abnormal mitochondrial fission and mitochondrial dysfunction-related DCM.

Despite the high abundance of fusion and fission regulatory proteins in the heart, mitochondria in adult cardiomyocytes exhibit static morphology and infrequent dynamic changes. These fusion and fission proteins may have functions beyond morphology regulation, or they may regulate cardiac function in DCM by regulating the mitochondrial dynamics of other cells in the heart, such as cardiac fibroblasts. As shown by Zhang et al. [56], they pointed to a novel noncanonical function of DRP1, in which DRP1 maintained or positively stimulated mitochondria respiration, biogenetics, and reactive oxygen species (ROS) signaling in adult cardiomyocytes, independent of morphological changes. In DCM, whether the fusion and fission proteins function through other mechanisms independent of mitochondrial dynamics remains to be elucidated.

### 3.3. MAMs Regulate Autophagy in DCM

Autophagy is an evolutionarily conserved lysosome-mediated degradation process that has fundamental roles in cellular homeostasis. Autophagosome and autolysosome formation are key processes in autophagy, which are mediated by autophagy-related genes (ATGs) [57, 58]. Autophagosome formation at MAMs in mammalian cells has been reported, with preautophagosome marker, autophagy-related 14-like, and the omegasome marker double FYV1 domain-containing protein1, localized in MAMs initiating autophagosome formation [59]. Another autophagosome formation marker, autophagy-related 2/5 (ATG2/5), also localizes at contact sites until the autophagy process is completed [59]. In addition, Beclin-1, a pro-autophagic protein localized in MAMs, plays a role in autophagosome formation and the autophagy process [60]. Inducing MAM dysfunction by knockout of MFN2 or PACS2 decreases the number of autophagosomes, and MFN2 deficiency impairs autophagosome-lysosome fusion [61]. Hu et al. have also shown that AMPK interacts directly with MFN2 to increase MAM numbers and induce autophagy [62]. Thus, these facts indicated that MAMs play key roles in the induction and execution of autophagy.

Autophagy is a double-edge sword, and basal autophagy is beneficial to the heart, whereas insufficient autophagy or excessive autophagy may promote pathological cardiomyopathy [63]. Previous studies reported that cardiac autophagy was suppressed in diabetes, accompanied by decrease in ATG5 and Beclin-1 expression levels [64]. However, the role of autophagy in DCM is controversial, as another study pointed to an increase in cardiac autophagy in type 2 diabetes through a Beclin-1-mediated pathway [65]. This discord in the findings is likely related to the unresolved question of whether an accumulation of autophagosomes in cells is the result of upregulation of autophagy or blockade of autophagic flux. As the autophagy process is highly dynamic, quantification of autophagy becomes a challenge [66]. It is important to consider not only the number of intracellular autophagosomes but also the autophagic degradative activity and autophagic flux. As reported previously, increased autophagic flux alleviates diabetes-induced cardiac injury [67]. Thus, autophagic flux insufficiency, resulting in maladaptive cardiac remodeling, should be considered a pivotal pathology in in DCM [68].

### 3.4. MAMs Regulate Inflammasome in DCM

The crucial role of inflammation in the pathogenesis of DCM is widely recognized. Inflammasomes comprise innate immune system receptors and sensors, which are activated in response to cellular stress and trigger the maturation of proinflammatory cytokines and the immune response [69]. To date, NOD-like receptor pyrin domain-containing 3 (NLRP3) is the sole inflammasome reported to be associated with MAMs, and it comprises NLRP3 protein, the adapter apoptosis-associated speck-like protein containing a C-terminal caspase recruitment domain (ASC) and pro-caspase-1. Under normal physiological conditions, NLRP3 is localized in the cytosol. When inflammasome is activated, the NLRP3 protein and its adaptor ASC are recruited to MAMs and are activated by MAM-derived effectors [70]. The NLRP3 inflammasome is activated by saturated fatty acids, ceramides, modified low density lipoprotein, and hyperglycemia in obesity and type 2 diabetes [71]. Continuous activation of inflammasomes ultimately leads to cardiac dysfunction [72]. In DCM, an early inflammatory response occurs as a protective mechanism against hyperglycemia. If hyperglycemia continues, a chronic inflammatory response will eventually lead to cardiomyocyte hypertrophy, apoptosis, and myocardial fibrosis [73].

The NLRP3 inflammasome recognizes signs of cellular stress, such as mitochondrial ROS production and Ca^2+^ signaling from damaged cells. Sustained influx of Ca^2+^ into the mitochondria via MAMs triggers mPTP opening, releasing risk-associated molecular patterns, finally leading to the activation of the NLRP3 inflammasom [70]. Yin et al. suggested that ROS could promote NLRP3 inflammasome activation [74]. Zhou et al. showed that mitophagy/autophagy blockade led to the accumulation of ROS, which activated the NLRP3 inflammasome [70]. Therefore, mitochondrial ROS and Ca^2+^ signaling pathways in MAMs are closely bound up with inflammasome activity. Based on the literature, Ca^2+^ communication between mitochondria and the ER may link MAMs to NLRP3 inflammasome activation in DCM. More research is needed to investigate the link of MAMs and inflammasome in DCM.

### 3.5. MAMs Regulate ER Stress in DCM

The ER plays a vital role in proteins folding. Disruption of ER homeostasis leads to an accumulation of misfolded proteins in the lumen, which triggers unfolded protein response (UPR) and ER stress. The stimulation of the UPR is sensed predominantly by three transmembrane proteins, protein kinase RNA-like endoplasmic reticulum kinase (PERK), inositol-requiring protein 1 (IRE1*α*), and activating transcription factor 6 (ATF6), which regulate the protein folding ability of ER [75]. Substantial evidence suggests that ER stress is a key mechanism in the development and progression of DCM. Continuous activation of the UPR mediates upregulation of apoptosis-related gene expression by affecting mitochondrial function, which eventually leads to apoptosis of cardiomyocytes and deterioration of cardiomyopathy.

IRE1*α* is expressed mainly in the MAMs, where it binds to the Sigma-1 receptor (Sig1R). Our previous study reported that the IRE1 pathway mediated the stimulatory effect of Sig1R on cardiac fibroblast activation [76]. The PERK signaling pathway plays a key role in ROS-mediated ER stress in DCM [77]. Previous research showed that MFN2 physically interacts with PERK and negatively regulates its activity [78]. Gao et al. indicated that with the aggravation of oxidative stress injury in DM, the cardiac MFN2 mRNA level decreased [79]. A recent study revealed that in high glucose (HG)-induced podocytes, HG activated the PERK pathway by downregulating MFN2 expression and reducing MFN2-PERK interaction [80]. Therefore, we speculate that the downregulation of MFN2 in cardiomyocytes under high glucose environment may lead to dissociation and continuous activation of PERK, which subsequently initiates UPR and ER stress.

## 4. MAMs Targeting as a Potential Therapeutic Strategy

### 4.1. Current Drugs in Treatment of DCM

Metformin is widely used to treat type 2 diabetes. A large-scale prospective study of 1,519 type 2 diabetes mellitus (T2DM) patients with heart failure indicated that treatment with metformin reduces cardiovascular mortality in this patient population [81]. In a preclinical study that examined the effect of metformin treatment for 4 months in type 1 diabetes mice, the authors observed clear improvements in the systolic and diastolic function of the heart [82].

The major mechanism of action of metformin is activation of AMPK [83]. In this review, we focus mainly on metformin's therapeutic effect on DCM through the MAMs proteins-related pathways. The NLRP3 inflammasome is activated in DCM, and AMPK inhibits NLRP3 expression by initiating autophagy. In DCM, metformin exerts cardioprotective and anti-inflammatory effects by activating AMPK autophagy pathway and inhibiting the NLRP3 inflammasome [53]. AKT and GSK3*β* are essential MAM components. Yang et al. showed that metformin ameliorates high glucose-induced cardiac damage through the AKT-GSK3*β* pathway in high glucose-exposed cardiomyocytes and animal models of diabetes [84]. Combined treatment with metformin and atorvastatin attenuated DCM by inhibiting oxidative stress and the impression of inflammation-related proteins, such as NLRP3, caspase-1, and 1L-1*β*. Combination therapy also restrained the apoptosis of cardiomyocytes by decreasing the expression of pro-apoptotic related proteins, including caspase-3 and BAX [85].

Sodium-glucose co-transporter 2 (SGLT2) inhibitors, such as empagliflozin, dapagliflozin, and canagliflozin, also serves as potential antidiabetic agents. In a cardiac magnetic resonance imaging study of changes in cardiac structure and function in 25 patients with T2DM, 6-month treatment with empagliflozin lowered left ventricle end diastolic volume compared to that in a control group [86]. Empagliflozin also suppressed oxidative stress and fibrosis by activating Nrf2/ARE signaling, as well as inactivating the TGF-*β*/SMAD pathway [87]. In 37 patients (25 males and 12 females) with T2DM, 32% of whom had pre-existing cardiovascular diseases, a 3-month treatment with canagliflozin significantly improved the left ventricular diastolic function [88]. Treatment with dapagliflozin for 6 months in 58 T2DM patients with stable heart failure appeared to have beneficial effects on left ventricle diastolic function [89]. Dapagliflozin reduced NLRP3/ASC inflammasome activation and activated AMPK both *in vivo* and *in vitro*. Attenuation of the NLRP3 inflammasome activation depends on AMPK activation [90]. In addition to activating AMPK, dapagliflozin has been identified activating mTOR [91]. Large-scale clinical trials aiming at investigating the impact of SGLT2 inhibitors on DCM are needed.

The glucagon-like peptide-1 receptor (GLP-1R) agonist is used to treat advanced stage T2DM. Preclinical studies have indicated that GLP-1R agonists, including exendin-4 and liraglutide, induce robust cardio protection [92, 93]. Exendin-4 relieves mitochondrial oxidative stress in diabetic heart. In models of T2DM models, the MFN1/MFN2 ratio was reduced in exendin-4-treated group [94]. Exendin-4 and liraglutide also protected cardiomyocytes under high glucose condition. High glucose incubation of cardiomyocytes significantly upregulated the expression of pro-apoptotic factor, but exendin and liraglutide abrogated this effect. These two drugs activate autophagy through the classical mTOR/ULK1-dependent pathway [95]. Younce et al. reported that exendin-4 attenuated high glucose-induced cardiomyocyte apoptosis in association with decreased ER stress and enhanced the activity of SERCA2a [96]. In addition, lipid regulation of GLP-1R agonists may improve diastolic function and attenuate diabetic cardiomyopathy. Wu et al. showed that exendin-4 improved the structure and function of diabetic hearts by inhibiting PPAR*α*-mediated lipid accumulation and toxicity regulated by the PKA/ROCK pathway [97].

Melatonin is widely used to treat insomnia and sleep disorders. Notably, the cardioprotective effects of melatonin have been described [98, 99]. Recent research confirms that melatonin acts as a regulator of MAMs. Application of melatonin inhibits IP3R, stabilizing the physical contacts between mitochondria and ER, and thus improving mitochondrial function and reducing cardiomyocytes damage. Moreover, some other MAM markers, Fis1, BAP31, and MFN2, were also inhibited by melatonin [100]. These data suggest that melatonin-induced cardioprotective effect is mediated via normalization of mitochondria-ER interaction.

### 4.2. Potential Targets

MFN2 was originally thought to be a mitochondrial protein that mediated OMM fusion. Recent research suggests that MFN2 appears to have a variety of roles in a nonfusion way. For example, MFN2 tether the ER to the mitochondria for Ca^2+^ signaling from the ER to mitochondria. MFN2 ablation or reduction increases the physical distance between mitochondria and the ER and disturb mitochondrial Ca^2+^ uptake [23, 44]. MFN2 is also involved in the cardiac autophagic process. Deficiency of MFN2 impairs autophagosome-lysosome fusion and leads to cardiac vulnerability and dysfunction [61]. Hu demonstrated that MFN2 expression is reduced in diabetic hearts, resulting in excessive mitochondrial fission, leading to mitochondrial dysfunction in DCM [48]. Reconstitution of MFN2 promotes mitochondrial fusion and alleviated mitochondrial dysfunction, consequently inhibiting the development of DCM [48]. Taken together, these findings suggest that regulation of MFN2 might be a potentially effective strategy for DCM treatment.

FUNDC1, an OMM protein, is highly conserved across species from drosophila to humans and highly expressed in cardiac muscle [26]. Diabetes increases FUNDC1 expression and aberrant MAM formation in cardiomyocytes, resulting in an increase in mitochondrial Ca^2+^ levels, mitochondrial dysfunction, and cardiac dysfunction. Cardiac-specific deletion of FUNDC1 improves mitochondrial function and attenuates cardiomyopathy in diabetic mice, confirming the causative role of FUNDC1 in this disorder [11]. However, there is some controversy regarding the role FUNDC1. According to Ren et al., FUNDC1 deficiency accentuated high fat diet-induced cardiac anomalies, including cardiac remodeling and intracellular Ca^2+^ mishandling [101]. The discord in the findings may be due to differences in the expression of FUNDC1 in different stages of DCM disease progression. As mentioned above, the development of DCM is a gradual process. During this process, it is likely that FUNDC1 expression may first increased and then decreased or vice versa. In conditions like DCM, where the disease progresses over time, the findings of these two studies may not necessarily be contradictory. Despite the discord in the findings, both studies prove that FUNDC1 is an extremely important regulator in DCM. FUNDC1 also interacts with OPA1 to coordinate mitochondrial fusion under normal conditions [46]. We speculate that extracellular hyperglycemia and metabolic dysregulation in DCM might reduce the interaction of OPA1 and FUNDC1, thereby increasing mitochondrial fragmentation. These findings collectively support the unique role of FUNDC1 as a powerful therapeutic target in DCM.

GSK3*β* is a multifunctional kinase. A fraction of GSK3*β* is localized to the MAM in the heart, where it specifically interacts with the IP3R Ca^2+^ channeling complex in MAMs. Apoptosis of cardiomyocytes in DCM involves overexpression of GSK3*β* [102]. Pharmacological or genetic inhibition of GSK3*β* may decrease the cardiomyocytes apoptosis of T2DM patients.

## 5. Conclusions

MAMs connect two important organelles (the ER and mitochondria), which have important roles in cellular functions. The importance of the contacts between the ER and mitochondria in the pathogenesis of DCM has been recognized. Several MAM-related proteins participate directly or indirectly in the regulation of the pathophysiological process of DCM via the regulation of lipids synthesis, insulin signaling, Ca^2+^ signaling, mitochondrial dynamics, ER stress, autophagy, and inflammation. Targeting MAMs could lead to the development of more efficient pharmacological approaches and potential biomarkers for the treatment of DCM.

Currently, various existing antidiabetic drugs, such as metformin, SGLT2 inhibitors, and GLP-1 agonists, provide significant cardiovascular protection in both animal models and patients with DCM, and these drugs represent the primary treatment options for patients with DCM. Several MAM proteins, such as MFN2, FUNDC1, and GSK3*β*, have been identified that play key roles in DCM. These proteins may have clinical applications as therapeutic targets and intervention strategies in DCM. In addition, several herbal compounds are known to regulate MAM and thus might have potential in DCM treatment. For example, Shengmai injection, a tradition Chinese herbal medicine extracted from Panax ginseng C.A. Mey., *Ophiopogon japonicus* (Thunb.) Ker Gawl., and *Schisandra chinensis* (Turcz.) Baill., can activate the AMPK signaling pathway [103]. As AMPK regulates FUNDC1 expression, targeting AMPK may provide a cardioprotective effect. Moreover, obacunone, a natural bioactive compound isolated from the Rutaceae family, downregulates the activity of GSK-3*β*, which may target on MAMs to stabilize the mitochondrial membrane potential [104]. Taken together, these findings provide strong evidence that MAMs may be the crucial target of DCM.

There are a number of unanswered questions surrounding the potential of MAMs as therapeutic targets in DCM. First, the pathways or mechanisms of some MAM proteins in DCM have not been clearly explained, even some contradictory views appeared in different researches. Second, the cell types and intervention methods in addition to the carriers, dosages, and targeted treatment sites differ from different researches. Third, current research is limited to *in vitro* cell experiments and animal experiments, and clinical studies are some ways off yet. Uniform research standards and intervention methods are necessary to further explore the functional mechanisms, clinical efficacy, and long-term effects of MAMs in DCM. In the future, more tether proteins will likely be identified between the ER and mitochondria. Further studies are required to shed light on how changes in activity of MAMs resident proteins contribute to the pathogenesis of DCM and how these proteins are expected to be clinically attractive therapeutic strategies.

## Figures and Tables

**Table 1:** Summary of the functional MAMs proteins.

Functions	MAMs proteins	Relevant functions in MAMs	Reference
Ca^2+^ transfer	IP3R1/2/3	Interacts with VDAC via GRP75, a major actor in ER Ca^2+^ release to mitochondria	[22]
	VDAC1	Acts as a Ca^2+^ uptake channel in the OMM	[22]
	GRP75	Chaperone protein connects IP3R and VDAC to form VDAC1/GRP75/IP3R1 channel complex	[22]
	PTPIP51	Interacts with VAPB at MAMs and regulates Ca^2+^ homeostasis	[24]
	VAPB	Interacts with PTPIP51 at MAMs and regulates Ca^2+^ homeostasis	[24]
	SERCA	Acts as an important pump involved in Ca^2+^ transport into ER	[33]
	Sig-1R	Generates a chaperone complex with BiP/GRP78 and prolongs Ca^2+^ signaling stabilizing subunit 3 of IP3R	[105]
	P53	Regulates SERCA activity and modulates ER-mitochondrial transfer	[106]
	PML	Regulates Ca^2+^ transfer and control apoptosis	[29]
	Calnexin	Interacts with SERCA, regulating Ca^2+^ transfer between contact sites	[107]
	Cytc	Interacts with IP3Rs and regulate Ca^2+^	[108]
	Bcl-2	Inhibit the opening of IP3Rs and downregulate IP3R-mediated Ca^2+^ flux	[109]
	CYPD	A partner of the IP3R1-GRP75-VDAC1 complex and changes the MAM spatial structure	[30]
	mTORC2	Regulates Ca^2+^ signaling by Akt regulation	[110]
	PP2A	Recruited by PML and inactivates AKT, facilitates IP3R-mediated Ca^2+^ release	[29]
	PTEN	PTEN regulates ER Ca2+ release through type 3 IP3R in a protein phosphatase-dependent manner	[111]
	Akt	Akt phosphorylates all IP3R isoforms and inhibits Ca^2+^ release from the ER	[38]
	GSK3*β*	Regulates organelle Ca^2+^ exchange	[37]
	FUNDC1	Binding of FUNDC1 to IP3R2 at the MAMs increases the Ca^2+^ concentration in both cytosol and mitochondrial matrix	[26]
	MFN2	Forms dimers with either MFN1 or MFN2 located on the mitochondria, controls the mitochondrial fusion	[23]
Mitochondrial dynamics	Bax	Interacts with MFN2 to promote mitochondrial fusion	[112]
	FUNDC1	Interacts with OPA1 to promote mitochondrial fusion; promote mitochondrial fission under hypoxic condition	[46]
	DRP1	Regulates mitochondrial fission	[18]
	INF2	Drives initial mitochondrial constriction	[50]
	MFF	Recruits DRP1 and regulates mitochondrial fission	[113]
	FIS1	Recruits DRP1 and regulates mitochondrial fission	[114]
	MiD49/51	Recruits DRP1 and regulates mitochondrial fission	[55]
Autophagy	ATG14L	Acts as preautophagosome marker, induces autophagosome formation	[59]
	ATG5	Acts as autophagosome marker	[59]
	PACS2	Knocking down PACS2 decreases the number of autophagosomes	[115]
	MFN2	Knocking down MFN2 decreases the number of autophagosomes	[115]
	VAPB	Regulates autophagy	[24]
	PTPIP51	Forms a complex with VAPB to regulate autophagy	[24]
	BECLIN1	Enhances the formation of MAMs and autophagosomes	[60]
	PINK1	Promote ER-mitochondrial tethering and autophagosome formation	[60]
Inflammation	NLRP3	NLRP3 inflammasome can be recruited to the MAM sites to sense mitochondrial damage	[70]
	ASC	The adaptor of NLRP3	[70]
	TXNIP	TXNIP activates NRLP3 inflammasome activation under mitochondrial oxidative stress conditions	[116]
ER stress	PERK	Induces apoptosis after ROS-based ER stress	[117]
	IRE1*α*	Responses to UPR stimulation; IRE1*α* ubiquitylation at MAM hinder ER-stress-induced apoptosis	[118]
	MFN2	Interacts with PERK and repress its activity	[78]
